# Supplementary material for: Awareness, Attitudes and Clinical Practices Regarding Human Papillomavirus Vaccination among General Practitioners and Pediatricians in Switzerland
Source: Vaccines (Basel). 2021 Apr 1;9(4):332. doi: 10.3390/vaccines9040332 (PMC8065954; doi:10.3390/vaccines9040332)
Supplement: Supplementary file 1 [file vaccines-09-00332-s001.pdf]

Table S1. Checklist for Reporting Results of Internet E-Surveys (adapted from [1]).

Item Category / Checklist Item	Explanation	Location in Paper
**Design**		
**Survey design**	Open web-based survey	Methods section
**Institutional Review Board approval and informed consent process**		
**IRB approval**	The local ethics committee of the canton of Zurich waived review and approval for this study, since the project lay outside the scope of the Federal Act on Research involving Human Beings (BASEC-Nr. Req-2020-00070)	Methods section
**Informed consent**	On the welcome page of the survey, all participants had to accept an informed consent statement. By selecting a corresponding tick box, respondents declared consent to participate and were forwarded to the survey questions. The statement contained the purpose of the study, gave information and contact data of the conducting institute, and revealed potential conflict of interests. All participants agreed that their data would be used for scientific analysis and published in an international peer reviewed journal	Methods section
**Data protection**	Guaranteed by the responsible researchers' personal login to the SurveyMonkey account with user name and password	Checklist only
**Development and pre-testing**		
**Development and testing**	The survey items were constructed by the research team and implemented with the SurveyMonkey web tool. Procedure and items of the survey were pre-tested, adapted and approved by a group of volunteering physicians affiliated with the responsible researchers' institute	Methods section and Checklist
**Recruitment process and description of the sample having access to the questionnaire**		
**Open survey versus closed survey**	Open survey.	Methods section
**Contact mode**	By email and mail	Methods section
**Advertising the survey**	None	Checklist only
**Survey administration**		
**Web/E-mail**	Web-based survey	Methods section

**Context**	The survey was designed using the commercial web tool SurveyMonkey (SurveyMonkey Inc., San Mateo, California, USA). The web survey appearance was neutral.	Checklist only
**Mandatory/voluntary**	Participation was voluntary.	Methods section
**Incentives**	All participants had the opportunity to take part in a lottery (one of 5 tablet computers).	Checklist only
**Time/Date**	The survey was open from 26.05.2020 to 24.09.2020	Methods section
**Randomization of items or questionnaires**	No	Checklist only
**Adaptive questioning**	Yes	Methods and results sections
**Number of Items**	40 (maximum)	Checklist only
**Number of screens (pages)**	17 (maximum)	Checklist only
**Completeness check**	All answers were voluntary. We included no completeness checks during the survey	Checklist only
**Review step**	Participants hat the option to switch between pages by using «go back to» and «proceed» buttons, and to change answers	Checklist only
**Response rates**		
**Unique site visitor**	Only participants or visitors completing at least the first page and proceeding to the next page were identifiable as respondents (total 463). Calculation of view and participation rate was therefore not possible	Results section
**Completion rate**	Completion was defined as filling in at least 75% of questions, leading to 422 included responses with a completion rate of 91%	Results section
**Preventing multiple entries from the same individual**		
**Cookies used**	Cookies were used for to assign unique identifiers to each client device and prevent double answers	Methods section
**Registration**	No login was required	Checklist only

Analysis		
**Handling of incomplete questionnaires**	Only questionnaires with at least 75% completed questions were analyzed. Descriptive statistics about missing data was provided	Methods section
**Questionnaires submitted with an atypical timestamp**	Time of access was recorded, no atypical timestamps were detected	Checklist only
**Statistical correction**	No statistical correction was implemented	Checklist only

Table S2. Results of the questionnaire section specific for the Swiss healthcare system. Abbreviations: GPs, general practitioners; HPV, human papillomavirus.

**Question**	**GPs** *n* **= 304 (72.0%)**	**Pediatricians** *n* **= 118 (28.0%)**	**Missing Data (% of total)**
**Is registered in the cantonal HPV vaccination program,** *n* **(%)**	196 (64.5)	111 (94.1)	0.0
**Questions directed at respondents registered in the cantonal HPV vaccination program** *n* = 307 (72.7% of total)			
**Sees any potential for improvement of the cantonal HPV vaccination program,** *n* **(%)**			0.0
No, the program is well-organized and the administrative burden is not too high	112 (57.1)	73 (65.8)	
Yes, general administrative burden is too high	35 (17.9)	16 (14.4)	
Yes, administrative burden linked with reimbursement is too high	36 (18.4)	8 (7.2)	
Yes, reimbursed amounts are too low	30 (15.3)	17 (15.3)	
Yes, for other reasons	12 (6.1)	11 (9.9)	
**Questions directed at respondents not registered in the cantonal HPV vaccination program** *n* = 115 (27.3% of total)			
**Reason for not being registered in the cantonal HPV vaccination program,** *n* **(%)**			0.0
I do not see enough potential candidates in my practice	49 (45.4)	0 (0.0)	
The general administrative burden is too high	24 (22.2)	2 (28.6)	
The administrative burden linked with reimbursement is too high	22 (20.4)	2(28.6)	
Reimbursed amounts are too low.	3 (2.8)	1 (14.3)	
Other	36 (33.3)	4 (57.1)	
**Aspects of the cantonal HPV vaccination program that should be changed in the first place for registration to be taken into consideration,** *n* **(%)**			0.0
The general administrative burden should be lower	44 (40.7)	3 (42.9)	
The administrative burden linked with reimbursement should be lower	27 (25.0)	2 (28.6)	
Reimbursed amounts should be higher	6 (5.6)	2 (28.6)	
I would not register in the HPV vaccination program regardless of circumstances	11 (10.2)	0 (0.0)	
Other	23 (22.2)	3 (42.9)	

References

1. Eysenbach, G. Improving the quality of Web surveys: the Checklist for Reporting Results of Internet E-Surveys (CHERRIES). *Journal of medical Internet research* **2004**, *6*, e34.
